# Bergmann's rule is followed at multiple stages of postembryonic development in a long‐distance migratory songbird

**DOI:** 10.1002/ece3.6721

**Published:** 2020-09-01

**Authors:** Joseph Youtz, Kelly D. Miller, Emerson K. Bowers, Samantha L. Rogers, Lesley P. Bulluck, Matthew Johnson, Brian D. Peer, Katie L. Percy, Erik I. Johnson, Elizabeth M. Ames, Christopher M. Tonra, Than J. Boves

**Affiliations:** ^1^ Department of Biological Sciences Arkansas State University State University Arkansas USA; ^2^ Department of Biological Sciences and Center for Biodiversity Research University of Memphis Memphis Tennessee USA; ^3^ Center for Environmental Studies Virginia Commonwealth University Richmond Virginia USA; ^4^ Integrative Life Sciences Doctoral Program Virginia Commonwealth University Richmond Virginia USA; ^5^ Audubon South Carolina National Audubon Society Harleyville South Carolina USA; ^6^ Department of Biological Sciences Western Illinois University Moline Illinois USA; ^7^ Audubon Louisiana National Audubon Society Baton Rouge Louisiana USA; ^8^ School of Environment and Natural Resources The Ohio State University Columbus Ohio USA

**Keywords:** body size, global change, heat conservation, ontogeny, prothonotary warbler, *Protonotaria citrea*

## Abstract

Bergmann’s rule is a well‐established, ecogeographical principle that states that body size varies positively with latitude, reflecting the thermoregulatory benefits of larger bodies as temperatures decline. However, this principle does not seem to easily apply to migratory species that are able to avoid the extreme temperatures during winter at higher latitudes. Further, little is known about the ontogeny of this relationship across life stages or how it is influenced by ongoing global climate change. To address these knowledge gaps, we assessed the contemporary relationship between latitude and body size in a long‐distance migratory species, the prothonotary warbler (Protonotaria citrea) across life stages (egg to adult) on their breeding grounds. We also measured historic eggs (1865‐1961) to assess if the relationship between latitude and size during this life stage has changed over time. In accordance with Bergmann’s rule, we found a positive relationship between latitude and body mass during all post‐embryonic life stages, from early nestling stage through adulthood. We observed this same predicted pattern with historic eggs, but contemporary eggs exhibited the reverse (negative) relationship. We suggest that these results indicate a genetic component to this pattern and speculate that selection for larger body size in altricial nestlings as latitude increases may possibly drive the pattern in migratory species as even rare extreme cold weather events may cause mortality during early life stages. Furthermore, the opposite relationships observed in eggs, dependent on time period, may be related to the rapidly warming environments of higher latitudes that is associated with climate change. Although it is unclear what mechanism(s) would allow for this recent reversal in eggs (but still allow for its maintenance in later life stages). This evidence of a reversal suggests that anthropogenic climate change may be in the process of altering one of the longest‐standing principles in ecology.

## Graphical abstract

Following Bergmann's rule, a positive relationship existed between latitude and body mass during all post‐embryonic developmental stages in a long‐distance migratory bird species, the prothonotary warbler (*Protonotaria citrea*). As this pattern appeared during the first days posthatching, these data suggest that the benefits of larger body size in colder environments may accumulate at an early age in altricial migratory species and may support the hypothesis of a genetic component underlying this pattern.

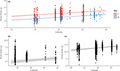

## 1 INTRODUCTION

It has long been recognized that the abiotic environment influences the evolution and distribution of organisms (Darwin, 1859; Olson et al., 2009). For endothermic organisms, climate is especially important as individuals are required to maintain their body temperature within a narrow thermal range to maintain homeostasis. One prevalent biogeographical pattern that has resulted from the understanding of this physiological necessity is the positive relationship between latitude and body size in endotherms, with the underlying explanation being the predictable differences in climate across latitudes (Bergmann, 1847; Blackburn, Gaston, & Loder, 1999; Rensch, 1938). This latitude‐body size cline, first described by K. Bergmann in 1847 and thus coined "Bergmann's rule," has been documented both inter and intraspecifically in a variety of endotherms, including many mammals and birds (Meiri & Dayan, 2003). The pattern occurs because larger organisms have a lower surface area:volume ratio which makes them more efficient at maintaining their body temperature and better able to survive in colder environments (James, 1970; Mayr, 1956; Yom‐Tov, 1993). Recently, this ecological “rule” has garnered renewed interest as global change has altered climate regimes across latitudes, often inconsistently across space (Gardner, Peters, Kearney, Joseph, & Heinsohn, 2011; Prokosch, Bernitz, Bernitz, Erni, & Altwegg, 2019; Teplitsky & Millien, 2013; Yom‐Tov, 2001).

Birds represent a taxon widely studied with respect to Bergmann's rule, and evidence suggests this pattern may be applicable to as many as 76% of bird species (Ashton, 2002). This pattern is not uniform across life history strategies, however, as nonmigratory species appear to express the pattern more often than migratory species, which is not surprising because migratory individuals can often avoid the coldest conditions, or associated food shortages, by moving to different latitudes during periods of thermal stress (Ashton, 2002; Rensch, 1936). The selective pressures that result in this latitude‐body size cline may be further reduced in migratory organisms because of their complex life histories, including different breeding and nonbreeding distributions, and because gene flow is rarely limited in these organisms due to their dispersal capabilities (Kimura et al., 2002; Kramer et al., 2018). Additionally, many migrants (e.g., Nearctic‐Neotropical avian species) typically only encounter temperate environments during a relatively short breeding season, which may not represent a strong enough selective pressure for this pattern to become established. Thus, because of this putative lack of a strong evolutionary mechanism, and the possibility that multiple mechanisms contribute to latitudinal variation in size (Blackburn et al., 1999), adherence to Bergmann's rule may be considered paradoxical in migratory species and has been understudied (but see Collins, Relyea, Blustein, & Badami, 2017; Gibson et al., 2019; Jones et al., 2005; Van Buskirk, Mulvihill, & Leberman, 2010).

It is possible that the perception of a lack of selective pressures that could result in the expression of Bergmann's rule in migratory species may be related to an overwhelming focus on adult organisms (e.g., Gibson et al., 2019; Husby, Hille, & Visser, 2011; Murphy, 1985). Selective forces related to thermoregulation (and hence body size) may in fact be strongest during juvenile stages, as these endotherms typically develop during specific thermal conditions (Salewski, Hochachka, & Fiedler, 2010). Altricial organisms, born naked and underdeveloped, do not develop endothermy for several days following hatching and their access to food is still constrained by parental feeding efficiency (i.e., while nest‐bound). For these organisms, climate may more strongly affect juvenile survival (Angilletta, Niewiarowski, Dunham, Leaché, & Porter, 2004; Bowers et al., 2016) or development (Andrew, Hurley, Mariette, & Griffith, 2017; Eeva, Lehikoinen, Rӧnkä, Lummaa, & Currie, 2002). This can be a limiting demographic rate for many organisms, particularly altricial birds (Dawson & Bortolotti, 2000; Griebel & Dawson, 2018; Ӧberg et al., 2015) potentially driving natural selection to a greater extent than selection on adult life stages. Furthermore, patterns that are established during earlier life stages may help to reinforce relationships during later life stages (Ardia, Pérez, & Clotfelter, 2010; Auer & Martin, 2017). Therefore, exploring Bergmann's rule across the full life cycle will improve our understanding of the mechanism(s) and adaptive benefit(s) driving this pattern, particularly in highly mobile, and migratory, endothermic taxa.

Another area of uncertainty with respect to Bergmann's rule relates to its future. Specifically, what will become of Bergmann's rule, one of the longest held ecological patterns, as global climate change proceeds? Anthropogenic climate change has already altered, and will continue to alter, the temperature regimes of regions of the Earth differentially (Rugenstein, Winton, Stouffer, Griffies, & Hallberg, 2013). While temperatures have increased across most of the planet, the rate of this increase has been strongly and positively related to latitude (Millennium Ecosystem Assessment, 2005). Thus, as climates at higher latitudes have changed faster than more tropical climates, we might predict that the latitude‐body size slope associated with Bergmann's rule may have already flattened and may continue to decrease over time eventually rendering Bergmann's rule obsolete. To explore this possibility, we can leverage the vast collections of historical specimens that have been curated in museums worldwide to improve our understanding of long‐term morphological change in a variety of taxa (Babin‐Fenske, Anand, & Alarie, 2008; Kirchman & Schneider, 2014; Meiri, Guy, Dayan, & Simberloff, 2009; Weeks et al., 2019).

To address these lingering questions related to Bergmann's rule, we used a model species, the prothonotary warbler (*Protonotaria citrea*). We selected this species for several reasons. First, it is a Nearctic‐Neotropical migrant that breeds across a relatively wide latitudinal range and readily nests within artificial cavities (i.e., nest boxes) that allow for unbiased access to all life stages (i.e., eggs, nestlings, and adults). Further, an active network of researchers maintains and monitors nest boxes throughout the breeding range, and recent work on this species suggests that most of the population winters in a relatively narrow range in Panama and Colombia (Tonra et al., 2019), thus removing the potential confounding variable of climatic variation on the wintering grounds. Finally, collection of eggs from this species occurred across its breeding range beginning in the mid‐19th century.

In this study, we first tested for adherence to Bergmann's rule in prothonotary warblers during multiple life stages (egg, young nestling, old nestling, and young and older adults). Then, using eggs, we compared patterns between historic and contemporary samples to assess how this pattern may have changed over time. This research furthers our understanding of why migratory species may exhibit this classic ecological pattern and provides further insight into the capacity for species to respond to our rapidly changing world.

## 2 MATERIALS AND METHODS

### 2.1 Study species

**FIGURE 1:**
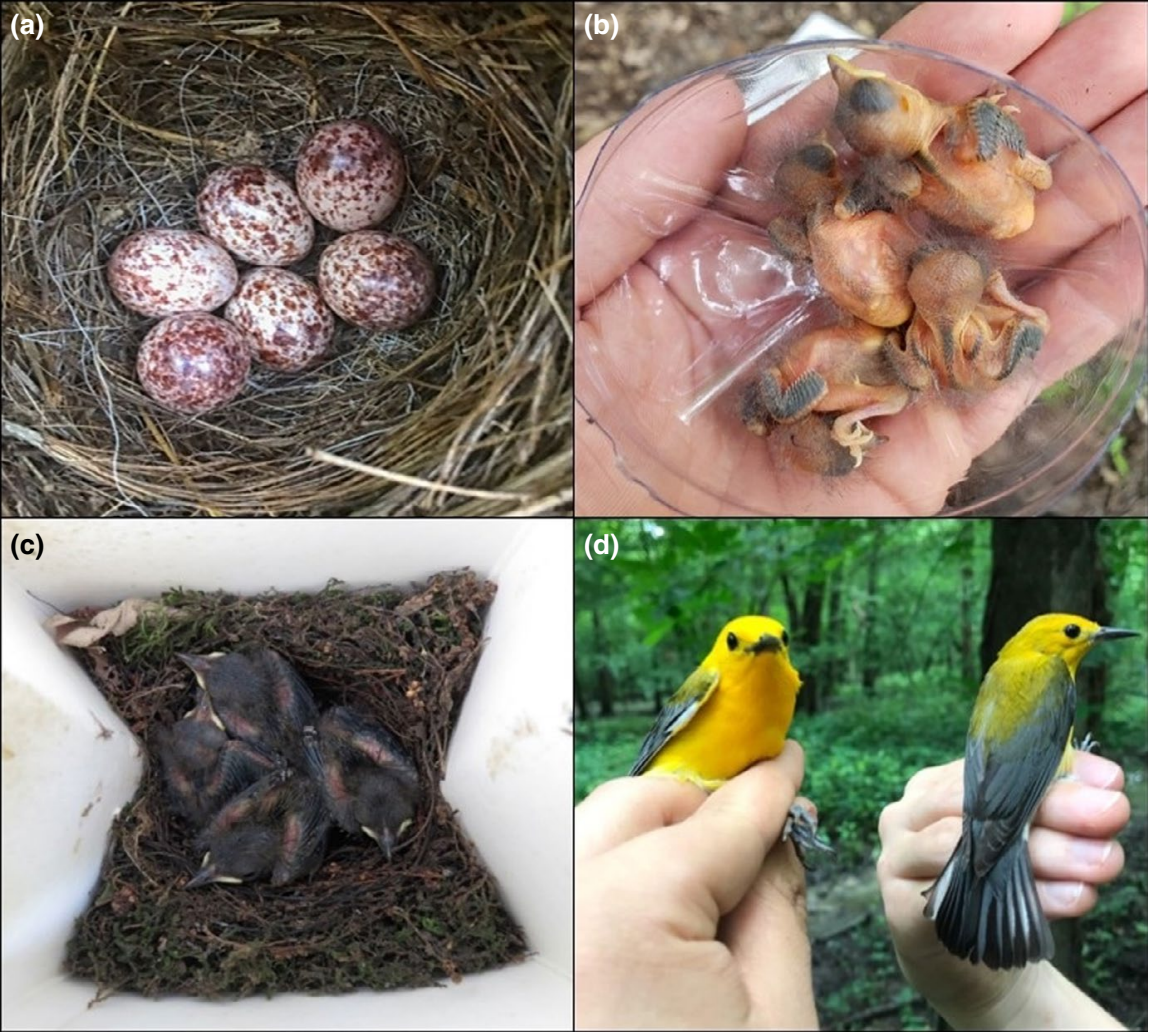
Prothonotary warblers (*Protonotaria citrea*) across life stages: (a) eggs, (b) early nestlings (3 days posthatching), (c) late nestlings (8 days posthatching), and (d) adults (male on left, female on right)

The prothonotary warbler is a Nearctic‐Neotropical migratory songbird that nests in secondary‐cavities (natural and artificial cavities, e.g., nest boxes) within riparian and bottomland hardwood forests of eastern and central North America, with most individuals breeding in the southeastern US (Petit, 1999; Slevin, Matthews, & Boves, 2018; Figure 1). Prothonotary warblers have an incubation period of 11–14 days, fledging occurs 9–11 days following hatching (Petit, 1999), and they can produce up to three broods in a season. Recent studies of geographic variation in the species (on the breeding grounds) have identified weak longitudinal genetic population structure (DeSaix et al., 2019) and variation in color and sexual signaling (Slevin, Bulluck, Matthews, & Boves, 2019). During the non‐breeding season, the bulk of the global population appears to winter in a relatively small area in Colombia and Panama (Reese et al., 2019; Tonra et al., 2019).

### 2.2 Study areas

**FIGURE 2:**
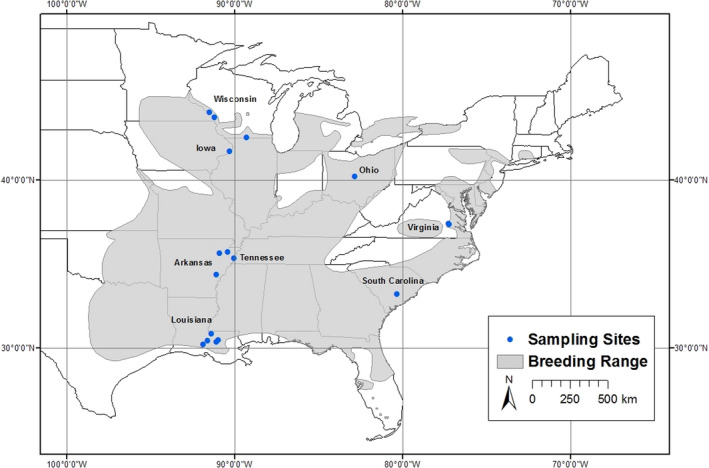
The breeding distribution of the prothonotary warbler (*Protonotaria citrea*light gray, Ridgely et al., 2003) and locations of field sites (blue dots) where contemporary eggs, nestlings, and adults were sampled during 2018–19

We studied prothonotary warblers across the eastern United States (Figure 2) during two breeding seasons (2018–19), following local breeding phenology; for example, we began monitoring them in late March in Louisiana, USA and later as birds moved northward (mid‐May in Wisconsin, USA). Study areas were in bottomland mixed hardwood, bald cypress (*Taxodium distichum*) forested swamps, or along riparian corridors. All sampled nests were built in artificial nest boxes that were made from either paper milk cartons, PVC, or wood.

### 2.3 Field methods

Upon hatching, we recorded individual nestling and brood mass at days 1–3 posthatching (“early nestling”) and at days 7–9 posthatching (“late nestling”). When possible, nestlings were aged based on known dates of hatching and, if not known, following the methods of Podlesak and Blem (2002). We also recorded sampling time and date to include as covariates in our statistical models. To reduce the handling time and potential effects of over‐handling at young ages, we did not record structural measurements from nestlings.

We captured adults using either playback of recorded territorial male song to lure them into a mist net or a hand‐held net placed directly over the nest box cavity during incubation or brooding. We determined sex using plumage coloration and age by molt limits according to Pyle (1997). For adult age classes, we categorized individuals as either second‐year (SY), which indicates that they were in their first breeding season, or after‐second‐year (ASY) which indicates that they were in at least their second breeding season. We measured relaxed wing chord, body mass, time of capture, date, and capture location coordinates. Other linear measurements (e.g., tarsus) were not included in this study, as wing chord has been shown to relate highly to passerine body size (Gosler, Greenwood, Baker, & Davidson, 1998) and interobserver variability within other metrics can be quite high (Goodenough, Stafford, Catlin‐Groves, Smith, & Hart, 2010). We banded each bird with an aluminum band (issued by the United States Geological Survey) and a unique combination of plastic color bands to later identify adults without recapture. Additionally, we leveraged data from previous studies (Slevin et al., 2018, 2019; Tonra et al., 2019) that included adults that were captured prior to the initiation of this study (2014–2018).

Once nests were active, we measured eggs after ≥ 3 eggs were present in the nest cup but prior to the initiation of incubation. From each active nest, we recorded clutch size and took digital photographs to measure egg dimensions in ImageJ (Schneider, Rasband, & Eliceiri, 2012). A subset of eggs were measured with calipers or a Mitutoyo QuickMini thickness gauge based on camera availability. All egg photograph measurements were made by a single individual (J. Youtz) to reduce potential observer bias. Additionally, we recorded the ambient temperature using a digital thermometer across all life stages of sampling to use as a covariate in our statistical models. From the field, we sampled and analyzed eggs from 29.8° to 42.5°N, early nestlings from 35.6° to 41.7°N, late nestlings from 29.8° to 41.7°N, and adults from 29.8° to 44.4°N.

### 2.4 Historic egg collections

To assess how the relationship between latitude and egg size has changed over time, we used eggs collected between 1865 and 1961 which were stored at various natural history museums: Peggy Notebaert Nature Museum‐Chicago (CHAS), Field Museum of Natural History (FMNH), Museum of Vertebrate Zoology (MVS), Florida Museum of Natural History (FLMNH), Smithsonian National Museum of Natural History (USNM), University of Arkansas Collections Facility (UAFMC), and the Cleveland Museum of Natural History (CMNH). We were unable to accurately estimate egg mass from these historical specimens, but because egg mass was correlated with egg dimensions in our contemporary sample (length: *R* ^2^ = .52, *p* < .001; width: *R* ^2^ = .65, *p* < .001), we used egg dimensions (obtained using ImageJ) as our measure of egg size in both contemporary and historical samples. From each historical clutch, we also recorded the collection location, date, and clutch size. All clutches lacking geographic coordinates were georeferenced using DeLorme Topo USA version 5.0 (DeLorme, 2004). Locations from which historic eggs were originally collected (Table A1) ranged from (28.5°N to 44.6°N latitude).

### 2.5 Data analyses

From our contemporary samples, we evaluated the relationship between latitude and egg/body size of individuals, by building linear mixed models with the package lme4 (Bates, Mächler, Bolker, & Walker, 2015) and function lmer in Program R (R Core Team, 2018). We modeled each developmental stage (egg, early nestling, late nestling, and adults) independently and qualitatively compared patterns across stages. First, we used body mass (or egg dimensions; length and width) as a continuous dependent variable, and treated latitude and longitude (to account for potential geographical differences as described by Slevin et al., 2018 and DeSaix et al., 2019), year, age (nestlings: days since hatching, adults: SY or ASY), and ambient temperature as independent fixed effects, and sampling time, date, and nest ID (to account for multiple eggs/nestlings in the same clutch or brood) as random effects (included in all models). For adult birds, we also included sex as a categorical fixed effect. For each life stage, we constructed all possible models that included all combinations of fixed effects (from univariate to global). We used Akaike's Information Criterion corrected for small sample size (AICc) to compare models and considered all models with ∆AICc ≤ 2 to be of equivalent fit (Burnham & Anderson, 2002). We employed the principal of parsimony when multiple models were equivalent. Once a final model was selected, we visually inspected residuals to ensure normality and homoscedasticity. We considered the body/egg size‐latitude relationship to be significant at *ɑ* = .05 and also examined the sign of the *β* coefficient to determine the directionality of this relationship and strength (if 95% confidence intervals did not overlap 0, we considered the relationship to be strong). We report the marginal (fixed effects) and conditional (total model) *R* ^2^ (Nakagawa & Schielzeth, 2013) for all final models using the package MuMIn (Bartoń, 2019) and function r.squaredGLMM. We also report 95% CIs of all coefficients included in top models to infer strength of relationships.

To assess how the latitude‐egg size relationship has changed over time, we used a dataset that included both historic and contemporary eggs along with three additional fixed effect variables: year as a continuous variable (coding from 1865 as “1” to 2019 as “154”) and a binary variable of time period (“historic” or “contemporary”), and the interaction between year × latitude. We then used the same model building process to evaluate whether any of these fixed effects significantly improved the model.

## 3 RESULTS

During 2018 and 2019, we measured 1,169 eggs, 306 early nestlings, and 983 late nestlings from 308 clutches, 71 (early nestling) broods, and 263 (late nestling) broods across the breeding range (Table A2). From 2014 to 2019, we measured 1,187 adult birds (645 males, 542 females, 329 SY, 673 ASY, 185 unknown age). Finally, we measured 261 historical eggs (from 55 clutches originally collected between 1865 and 1961) laid across much of the same breeding distribution where contemporary sampling occurred.

### 3.1 Nestlings

**FIGURE 3:**
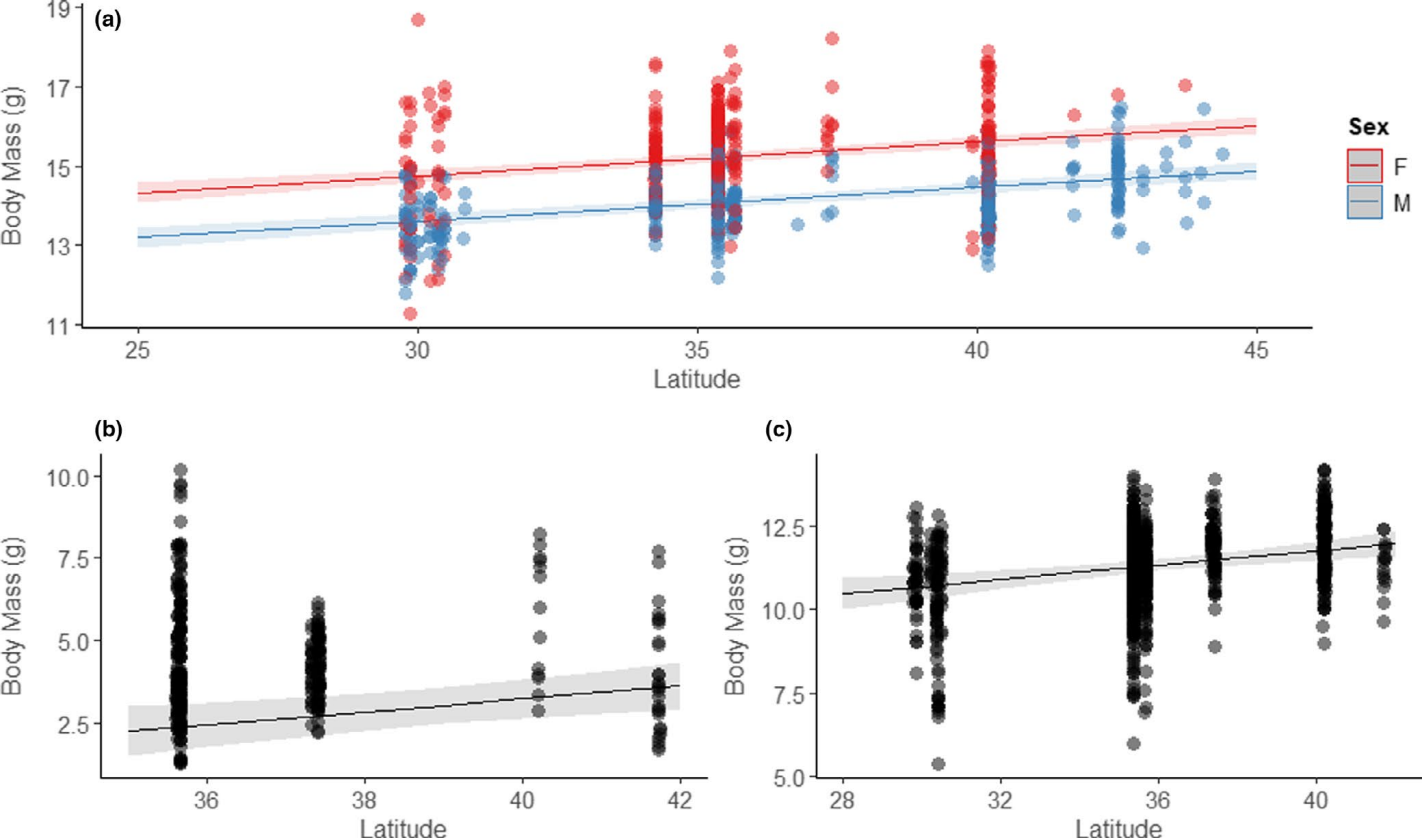
Relationships between breeding latitude and body mass for (a) adult (males in blue, females in red), (b) early nestling (1–3 days posthatching), and (c) late nestling (7–9 days posthatching) prothonotary warblers (*Protonotaria citrea*). Scatter points display the observed data and regression lines show the fitted line from the final models, along with 95% confidence intervals

The top model explaining early nestling (1–3 days posthatching) mass included the fixed effects of latitude, temperature, and nestling age (for random effects; see methods, Table 1, Table A3). Latitude was positively associated with body mass (marginal *R* ^2^ = .62, conditional *R* ^2^ = .84, *β* = 0.24, 95% CI: 0.1 to 0.38, *p* = .002), while the CI of temperature overlapped 0. This equates to an increase of 0.25 g of body mass/° latitude (Figure 3b.). The top model explaining late nestling (7–9 days posthatching) mass included the fixed effects of latitude, longitude, temperature, and age. Once again, latitude was positively associated with body mass (marginal *R* ^2^ = .18, conditional *R* ^2^ = .66, *β* = 0.28, 95% CI: 0.14–0.43, *p* < .001) which equates to an increase of 0.11 g of body mass/° latitude (Figure 3c.). Longitude correlated similarly with body size (*β* = 0.16, 95% CI: 0.02–0.3, *p* = .03), while the CIs for temperature and age both overlapped 0. At the latitudinal extremes, early nestlings from the farthest northern broods were on average 14.7% heavier than those from the farthest southern broods. Some of the latitudinal size differential was lost during development, but late nestlings from the farthest northern broods remained, on average, 7.5% heavier than those from the farthest southern broods.

**TABLE 1:** Top models (and nulls) describing the relationship between sampling latitude and size in prothonotary warblers (*Protonotaria citrea*) across egg (historic and contemporary), nestling (“early” and “late”), and adult life stages

Developmental stage	Model fixed effects	AICc	∆AICc	Weight
‘Early’ nestlings	Latitude + Temperature + Age	747.39	0.00	0.44
	Latitude + Longitude + Temperature + Age	748.29	0.9	0.28
	Latitude + Year + Temperature + Age	749.24	1.85	0.18
	Null	1,397.42	650.03	<0.01
‘Late’ nestlings	Latitude + Longitude + Temperature + Age	2,620.22	0.00	0.31
	Latitude + Longitude + Age	2,620.38	0.16	0.29
	Null	2,669.45	49.07	<0.01
Adults	Latitude + Sex + Age	1,965.85	0.00	0.42
	Latitude + Longitude + Year + Sex + Age	1,967.52	1.67	0.18
	Null	2,227.5	261.65	<0.01
Historic egg width	Latitude × Year	531.35	0.00	0.61
	Latitude	532.54	1.19	0.33
	Null	555.28	23.93	<0.01
Historic egg length	Latitude × Year	573.43	0.00	0.9
	Null	612.22	38.79	<0.01
Contemporary egg width	Latitude + Longitude	1,192.26	0.00	0.43
	Latitude + Longitude + Temperature	1,193.12	0.86	0.28
	Null	1,201.14	8.88	0.01
Contemporary egg length	Latitude + Longitude	1,731.57	0.00	0.6
	Null	1,735.36	3.79	0.09

### 3.2 Adults

The top model explaining adult body mass included the fixed effects of latitude, sex, and age (along with the random effects of date and time). Latitude was again positively associated with body mass (marginal *R* ^2^ = .31, conditional *R* ^2^ = .42, *β* = 0.29; 95% CI: 0.22–0.36, *p* < .001) which equated to an increase of 0.09 g of body mass/° latitude (Figure 3a.). Between sexes, males weighed, on average, 1.17 ± 0.07 g less than females. ASY birds weighed on average, 0.26 ± 0.08 g more than SY birds.

### 3.3 Eggs: historic

**FIGURE 4:**
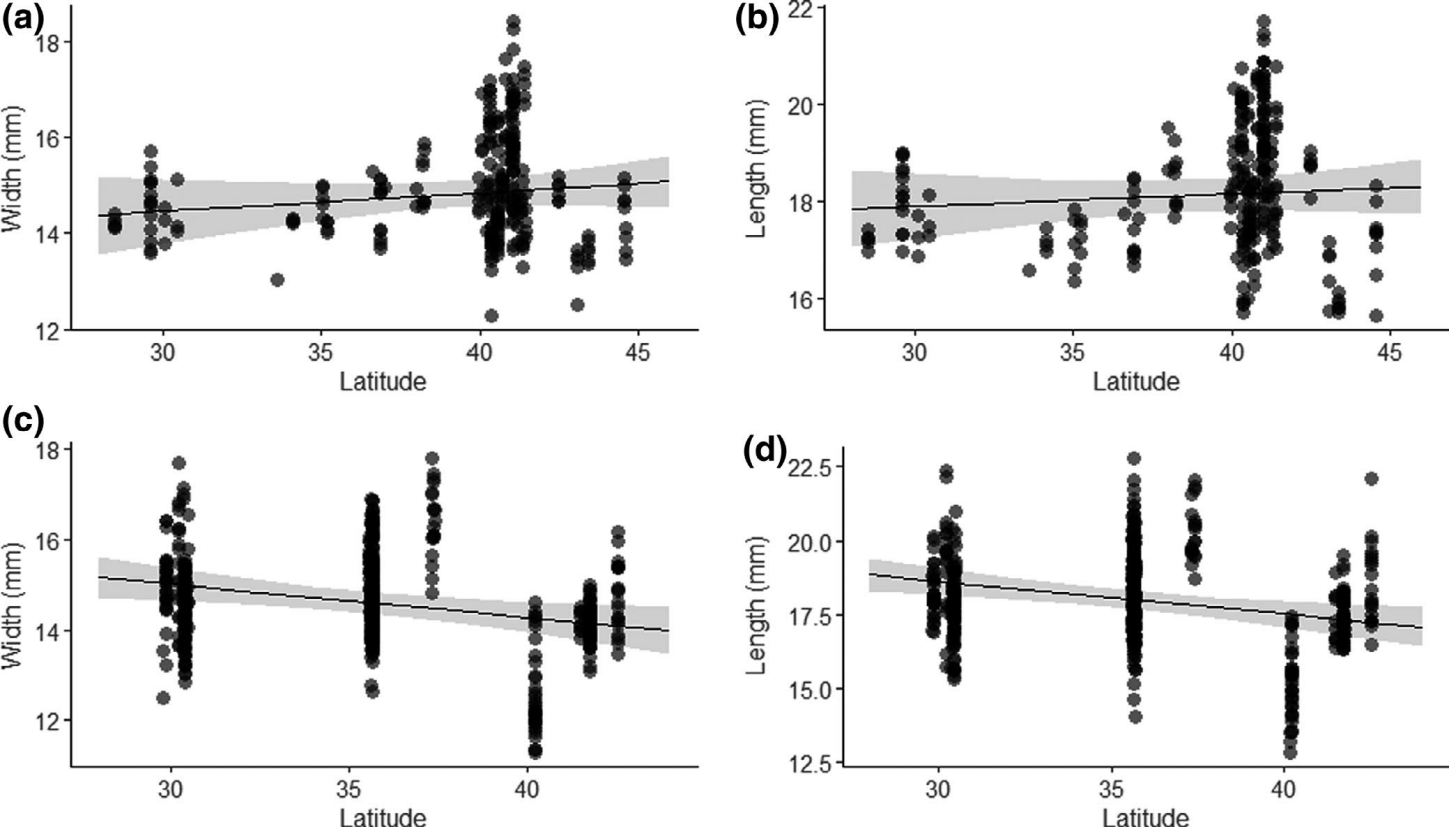
Relationships between breeding latitude and (a) historic egg (collected between 1865 and 1961) width and (b) length and (c) contemporary egg (collected 2018–19) width and (d) length for prothonotary warblers (*Protonotaria citrea*). Scatter points display the observed data and regression lines show the fitted line from the final models, along with 95% confidence intervals

The top models explaining historical egg size (width and length) included the fixed effect of the interaction between year and latitude (along with the random effects of date and nest ID). As seen with nestlings and adults, latitude was positively associated with historic egg dimensions. It was positively related to egg width (marginal *R* ^2^ = .06, conditional *R* ^2^ = .78, *β* = 0.11, 95% CI: −0.13 to 0.3, *p* = .03) with an increase of 0.02 mm/° latitude increase for width (Figure 4a.). Similarly, latitude was positively associated with historic egg length (marginal *R* ^2^ = .13, conditional *R* ^2^ = .81, *β* = 0.12, 95% CI: −0.1 to 0.35, *p* = .001), with an increase of 0.04 mm/° latitude increase in length (Figure 4b.).

### 3.4 Eggs: contemporary

The final model explaining contemporary egg width included the fixed effects of latitude and longitude (along with the random effects of nest ID and time). In this case, latitude was negatively associated with contemporary egg width (marginal *R* ^2^ = .09, conditional *R* ^2^ = .87, *β* = −0.37, 95% CI: −0.55 to −0.19, *p* < .001, Figure 4c.), which equated to a decrease of 0.1 mm/° latitude in width. From this model, longitude was positively associated with contemporary egg width (*β* = 0.29, 95% CI: 0.17–0.42, *p* < .001). The final model explaining contemporary egg length also included the fixed effects of latitude and longitude. Latitude was also negatively associated with egg length (marginal *R* ^2^ = .07, conditional *R* ^2^ = .76, *β* = −0.3, 95% CI: −0.47 to −0.14, *p* < .001, Figure 4d.) which equated to a 0.11 mm/° latitude decrease in length. Similarly, longitude was positively associated with contemporary egg length (*β* = 0.23, 95% CI: 0.11–0.35, *p* < .001).

## 4 DISCUSSION

Bergmann's rule remains one of the most widely recognized ecological principles and is often considered an explanation for spatial variation in body size for numerous taxa. However, studies of this pattern have typically focused on adults and sedentary organisms that span a broad latitudinal distribution. In addition, there remains considerable uncertainty about how global climate change will affect this long‐standing principle. Here, we leveraged a network of contemporary box‐nesting populations along with museum egg collections to evaluate Bergmann's rule across the ontogeny of, and over time in, a long‐distance avian migrant. As predicted by Bergmann's rule, we found a positive relationship between breeding latitude and body mass in multiple developmental stages of prothonotary warblers. The cline was present on the breeding grounds in the youngest hatchlings and remained through adulthood. However, we observed a reversal in the pattern between latitude and egg size within our contemporary egg samples. Latitude was positively associated with historic egg dimensions, yet we observed the reverse relationship with respect to egg size and latitude in contemporary egg samples. This apparent recent change may reflect a response to differential temperature changes across latitudes over the past century or other factors that have yet to be explored.

Previous tests of Bergmann's rule have come to inconsistent conclusions when focusing on migratory organisms, with some taxa displaying weak or no adherence (Ashton, 2002; Collins et al., 2017) and others exhibiting adherence across breeding (e.g., Jones et al., 2005) and nonbreeding distributions (e.g., Gibson et al., 2019). The occurrence of the latitude‐body mass cline nearly immediately after hatching that we observed in prothonotary warblers provides a unique perspective that may help explain some of these inconsistencies observed in migratory species. Particularly among temperate‐tropical long‐distance migrants, adults encounter very different thermal conditions throughout their annual cycle, and the heat conservation benefits associated with Bergmann's rule may be minimal as they escape temperate regions during the coldest periods. Thus, for this pattern to reflect a thermoregulatory mechanism, it would seem that individuals must be influenced by relatively subtle differences in temperature across their breeding distribution. For example, in our study daily low temperatures at our northern sites at arrival (early May in WI) ranged from 0.6 to 15.6°C while low temperatures at our southern sites (late March in coastal LA) ranged from 3.3 to 20.6°C. Thus, it seems somewhat unlikely for selection to act on adult birds (that possess insulative feathers) when facing such minor differences in temperature. However, it seems reasonable to expect that altricial nestlings, which have yet to develop insulative feathers, to face much stronger selection related to thermoregulation. Most altricial songbirds lack downy feathers until ~6 days old and even modest ambient temperature declines (and related body temperature declines) could be a major source of mortality. In fact, temperature‐related nest failure and nestling mortality have been documented in temperate breeding birds such as tree swallows (*Tachycineta bicolor*, Gentes, Waldner, Papp, & Smits, 2006) and bull‐headed shrike (*Lanius bucephalus*, Takagi, 2001). Anecdotally, we have observed cerulean warblers (*Setophaga cerulea*) and prothonotary warblers suffer egg/nestling mortality following extreme cold weather (below 0°C; T. J. Boves, unpublished data; L. P. Bulluck, unpublished data). Additionally, even if nestlings are not killed by cold weather events, low temperatures can limit prey availability (Jenni‐Eiermann, Glaus, Grüebler, Schwabl, & Jenni, 2008) and have long‐term negative effects on nestling development (Ardia et al., 2010; Eeva et al., 2002), bolstering the selective pressure for larger nestling body size at high latitudes.

Another climate‐related (and nonexclusive) explanation for the existence of this pattern in prothonotary warblers could be the constraint of heat stress. High ambient temperatures during development in the south may actually limit nestling growth and lead to reduced nestling size in the warmest areas (Andrew et al., 2017). Further, these size reductions may carry over to the adult stage (Andrew, Awasthy, Griffith, Nakagawa, & Griffith, 2018), suggesting that these consequences could be important for maintaining this pattern during multiple life stages. This plasticity of body size in response to environmental conditions may offer an underappreciated mechanism related to Bergmann's rule and deserves further exploration. However, this still remains a somewhat tenuous explanation, as other species have shown *increases* in body size in response to higher temperatures (e.g., Gardner et al., 2013) suggesting that a high temperature threshold likely exists, and impacts of increased temperature on avian body size are dynamic and context dependent.

**FIGURE A1:**
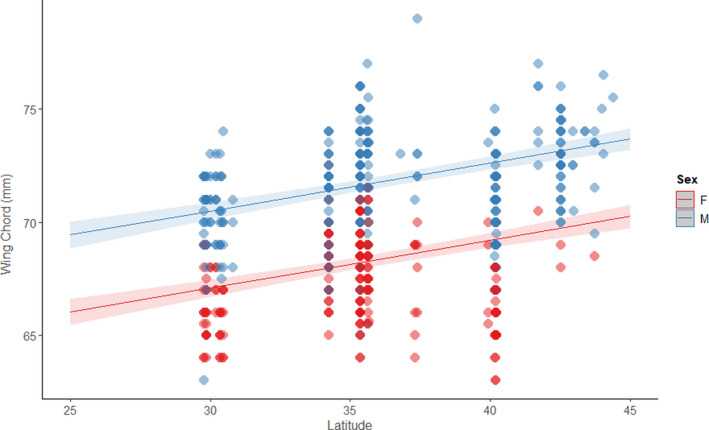
Relationships between adult wing chord length and breeding latitude for prothonotary warblers (*Protonotaria citrea*) (marginal *R* ^2^ = .58, conditional *R* ^2^ = .61, *β* = 0.28; 95% CI = 0.22–0.35; *p* < .001). The final model included latitude, longitude, sex, and age as fixed effects. Scatter points display the observed individual data (males in blue, females in red) and regression lines show the fitted line from the final model, along with 95% confidence intervals

Although it is possible that adult adherence to this pattern is simply a neutral byproduct of early developmental selection with no adaptive value at later stages, we suggest that the adaptive benefits associated with this pattern are not limited to a single life stage. In addition to some benefit to adults related to thermoregulation in areas of high climatic variability (which is typically greater at higher latitudes, Serreze et al., 2000), the pattern in adults could also be related to variation in migratory distances among local breeding populations. For example, if warblers from northern breeding sites have to complete longer migratory journeys, this could favor larger adult body size (assuming larger bodies are beneficial to completing these longer journeys). In fact, prothonotary warblers exhibit low migratory connectivity between breeding and wintering grounds, with much of the wintering population concentrated in Colombia (Bulluck et al., 2019; Tonra et al., 2019), so breeding latitude is likely correlated with migratory distance. Additionally, Hoover (2003) found that adult individuals within this species displayed high breeding site fidelity, such that one could predict that body size (and subsequently wing chord length, Figure A1) is associated with breeding locality. Finally, emigration and natal dispersal for this system are thought to be relatively low (McKim‐Louder, Hoover, Benson, & Schelsky, 2013), potentially reinforcing the establishment of these traits within recruited individuals. Future studies that examine return rates of both hatch‐year individuals and older adults of varying sizes across latitudes and a comparison of our results to species that exhibit greater migratory connectivity may help elucidate this possibility.

The mechanism(s) driving the patterns we observed could include both genetics and resource availability. As body size is strongly related to important traits such as thermoregulatory (especially at such early post‐embryonic stages) and migratory ability, it would seem likely that there is at least some genetic basis for these patterns. However, a recent study of the population genomics of prothonotary warblers found little latitudinal population structure (but greater longitudinal variation, possibly related to geographic isolation between migratory pathways; DeSaix et al., 2019). This genomic work, however, was not designed to detect genetic differences related to body mass variation. It is also unclear how variation in resource availability on the breeding grounds could contribute to the pattern, but increased prey availability could further advance this clinal pattern (Johnston, 1993). This may be particularly important within early nestlings, who display the pattern following hatching but rapidly develop a stronger latitude‐mass association as they age. This could suggest that the initial expression of the pattern has a genetic basis but is reinforced by provisioning and resource investment from adult birds. It may also be that larger individuals are able to minimize their likelihood of starvation during low resource periods at high latitudes (Goodman, Lebuhn, Seavy, Gardali, & Bluso‐Demers, 2012); however, this seems unlikely to explain the cline in nestling birds who store little, if any, energy and require constant feeding to continue their development (Ricklefs, 1983). To better understand how both genetic and resource variation contribute to this relationship, latitudinal studies of resource availability and nestling provisioning along with studies that attempt to identify the genetic basis of body size variation, in this and other species, are warranted.

Avian eggs require incubation temperatures of 36–39°C to maintain embryo viability (Webb, 1987) and large eggs may offset heat loss during cold periods, so we expected eggs to follow the same positive latitude‐size relationship we observed in nestling and adult prothonotary warblers. We did observe a consistent positive relationship between latitude and size across postembryonic life stages and in historic eggs, but we did not observe the same relationships in contemporary eggs. In fact, we documented a *negative* relationship between latitude and size in contemporary prothonotary warbler eggs. Taken alone, this contemporary negative relationship could be related to the fact that clutch sizes increase with latitude in this species (Petit, 1999) and recent increases in climatic variability in the northern portions of their range may have altered the number of days below freezing during the breeding period. Thus, prothonotary warblers may adaptively lay smaller eggs in colder environments to allow for improved incubation efficiency and to counteract heat loss related to egg size, especially when there are more eggs to incubate (Kim, Park, Choy, Ahn, & Yoo, 2009; Niizuma, Takagi, Senda, Chochi, & Watanuki, 2005; Reid, Monaghan, & Ruxton, 2000). However, given that we also observed a *positive* relationship between latitude and early nestling size, the paradoxical question of how smaller eggs could produce larger nestlings remains. Although speculative at this point, the answer may lie in the chemical composition of the egg itself. As yolk provides the majority of nutrition (Vleck & Hoyt, 2004), it is possible that it comprises a greater proportion of the egg at higher latitudes. Although interspecific variation in avian egg size has been relatively well studied (Christians, 2002; Sotherland & Rahn, 1986) little is known about the intraspecific variation of this phase of the reproductive cycle. Currently, research on the relationship between egg size and nestling development typically suggests that there is a positive relationship (Pinowska et al., 2004) that becomes less pronounced as individuals age (Smith & Bruun, 1998). But no research, to our knowledge, exists regarding how egg composition (e.g., yolk content), independent of egg size, affects nestling size and development, thus research in this area is still required.

As higher latitudes have warmed decisively faster than lower latitudes (and will likely continue to; Intergovernmental Panel on Climate Change, 2018; Rugenstein et al., 2013), the benefits of laying larger eggs at higher latitudes may have recently been reduced, resulting in the reversal of the cline we observed in contemporary eggs. Although this cline reversal in eggs may in fact reflect egg‐specific adaptations related to differentially changing climates, it is purely speculative at this point. However, Tryjanowski, Sparks, Kuczynski, and Kuzniak (2004) found that red‐backed shrike (*Lanius collurio*) egg size declined over a period of more than 30 years (while springtime temperatures increased), suggesting that this similar phenomenon may occur and is unlikely to be limited to prothonotary warblers. It could also be (at least partially) a by‐product of a putative concurrent softening of the cline in adult (esp. female) body size over the same time period (as smaller individuals typically lay smaller eggs, González‐Solis, Becker, Jover, & Ruiz, 2004). It would be feasible to test this hypothesis by replicating our egg analysis using historic museum specimens of adults collected from across breeding range (using structural measurements, such as tarsus, and comparing with contemporary samples). Recently, Weeks et al., (2019) used museum specimens to document large‐scale reductions in adult body size in many species of birds over time (and attributed this to adaption to climate change), but no one has yet assessed if this reduction has occurred more rapidly in northern species or populations. It is also possible that as egg size has changed over time, nest structural characteristics (Mainwaring et al., 2012) or clutch size (Laaksonen, Ahola, Eeva, Väisänen, & Lehikoinen, 2006) might have also changed, resulting in a complicated adaptive landscape involving multiple life history traits. Future studies should be designed to explore how these factors may interact to potentially result in this relaxation or reversal of Bergmann's rule and to test the generality of our results using other species.

## 5 CONCLUSION

We documented a positive relationship between latitude and body size following Bergmann's rule across multiple life stages, beginning at the earliest postembryonic stage, in a long‐distance migratory species. The early life‐stage manifestation of this pattern suggests at least some genetic basis and may be explained by the sensitivity of young (altricial) nestlings to extreme cold weather events, which were historically more likely to occur as latitude increases (even in temperate spring). The pattern may also be, at least partially, explained by the plasticity of this trait due to environmental influences (such as reduced nestling development under high temperatures). Our study did not explicitly test the mechanisms (e.g., variation in genetics, food resources, or egg composition) proximately driving this relationship and future studies to specifically test these mechanisms are warranted. Using museum specimens, we found that, historically, eggs also followed this classic pattern, as thermoregulatory benefits of larger size may also likely apply to eggs. However, these benefits may be relatively less important, as we showed that the relationship between latitude and egg size reversed in contemporary samples. We speculate that this reversal may be related to differential climate change across latitudes which has altered the benefits of laying larger eggs at higher latitudes, but more research is necessary to assess this hypothesis. This reversal of the classic eco‐biogeographical principle further supports data that global climate change is altering selection pressures and leading to alterations in the body sizes of endothermic organisms (Brommer, Hanski, Kekkonen, & Väisänen, 2015; Prokosch et al., 2019; Yom‐Tov, 2001, Weeks et al., 2019).

## CONFLICT OF INTEREST

We declare no conflict of interest.

## AUTHOR CONTRIBUTIONS

**Joseph Youtz:** Conceptualization (lead); data curation (equal); formal analysis (lead); funding acquisition (equal); investigation (equal); writing‐original draft (lead). **Kelly D. Miller:** Data curation (equal); writing‐review & editing (equal). **Emerson K. Bowers:** Data curation (equal); supervision (supporting); writing‐review & editing (equal). **Samantha Rogers:** Data curation (equal); investigation (equal). **Lesley P. Bulluck:** Investigation (equal); supervision (supporting); writing‐review & editing (equal). **Matthew Johnson:** Data curation (equal); investigation (equal). **Brian D. Peer:** Data curation (equal); funding acquisition (equal); supervision (supporting); writing‐review & editing (equal). **Katie L. Percy:** Data curation (equal); investigation (equal); writing‐review & editing (equal). **Erik I. Johnson:** Investigation (equal); writing‐review & editing (equal). **Elizabeth M. Ames:** Data curation (equal); investigation (equal); writing‐review & editing (equal). **Christopher M. Tonra:** Supervision (supporting); writing‐review & editing (equal). **Than J. Boves:** Conceptualization (lead); funding acquisition (equal); supervision (lead); writing‐review & editing (lead).

## Data Availability

Data available from the Dryad Digital Repository https://doi.org/10.5061/dryad.5mkkwh73x.

## APPENDIX A

**TABLE A1:** The number of prothonotary warbler natural history museum egg clutches (*n*) measured to test for Bergmann's rule, displayed by the state of collection

Collection locality	*n*
Arkansas	3
Florida	6
Illinois	27
Indiana	3
Iowa	2
Kansas	3
Michigan	2
Minnesota	2
Missouri	1
Ohio	2
Oklahoma	2
Virginia	2

**TABLE A2:** The number of prothonotary warbler sample types (*n*) measured to test for Bergmann's rule

Breeding locality	Eggs (*n*)	“Early” nestlings (*n*)	“Late” nestlings (*n*)	Adults (*n*)
Arkansas	63	40	30	161, 79 M, 82 F
Iowa	20	8	6	8, 6 M, 2 F
Louisiana	50	0	38	338, 193 M, 145 F
Missouri	0	0	0	1, 1 M, 0 F
Ohio	12	3	54	199, 122 M, 77F
South Carolina	0	0	0	34, 22 M, 12 F
Tennessee	138	0	115	289, 145 M, 144 F
Virginia	20	20	20	99, 22 M, 77 F
Wisconsin	5	0	0	58, 55 M, 3 F

Note

Eggs (*n* = clutches) and nestlings (*n* = broods) were measured from 2018 to 2019 and adult birds were measured from 2014 to 2019 and are displayed as the total number of individuals and the total number from each sex sampled

**TABLE A3:** Models (and nulls) describing the relationship between sampling latitude and size in prothonotary warblers (*Protonotaria citrea*) across egg (historic and contemporary), nestling (“early” and “late”), and adult life stages

Developmental stage	Model fixed effects	AICc	∆AICc	Weight
‘Early’ nestlings	Latitude + Temperature + Age	747.39	0.00	0.44
	Latitude + Longitude + Temperature + Age	748.29	0.9	0.28
	Latitude + Year + Temperature + Age	749.24	1.85	0.18
	Latitude + Longitude + Year + Temperature + Age	750.43	3.04	0.1
	Latitude + Longitude + Year + Age	771.7	24.31	<0.01
	Latitude + Age	808.36	60.97	<0.01
	Latitude + Longitude + Age	808.47	61.08	<0.01
	Latitude + Temperature	845.28	97.89	<0.01
	Latitude + Longitude + Year + Temperature	847.45	100.06	<0.01
	Latitude	921.5	174.11	<0.01
	Null	1,397.42	650.03	<0.01
‘Late’ nestlings	Latitude + Longitude + Temperature + Age	2,620.22	0.00	0.31
	Latitude + Longitude + Age	2,620.38	0.16	0.29
	Latitude + Longitude + Year + Temperature + Age	2,622.23	2.01	0.11
	Latitude + Longitude + Year + Age	2,622.25	2.03	0.11
	Latitude + Age	2,622.97	2.75	0.08
	Latitude + Temperature + Age	2,623.2	2.98	0.07
	Latitude + Longitude	2,627.14	6.92	0.01
	Latitude	2,628.97	8.75	<0.01
	Latitude + Longitude + Year	2,629.07	8.85	<0.01
	Latitude + Temperature	2,630.34	10.12	<0.01
	Latitude + Year + Temperature	2,632.37	12.15	<0.01
	Longitude + Year + Temperature	2,641.36	21.14	<0.01
	Null	2,669.45	49.07	<0.01
Adults	Latitude + Sex + Age	1965.85	0.00	0.42
	Latitude + Longitude + Year + Sex + Age	1967.52	1.67	0.18
	Latitude + Longitude + Temperature + Sex + Age	1968.16	2.31	0.13
	Latitude + Year + Temperature + Sex + Age	1968.62	2.77	0.11
	Latitude + Longitude + Year + Temperature + Sex + Age	1969.18	3.33	0.08
	Latitude + Longitude + Sex	1970.37	4.52	0.04
	Latitude + Sex	1971.88	6.03	0.02
	Latitude + Longitude + Year + Temperature + Sex	1972.35	6.5	0.02
	Longitude + Year + Temperature + Sex + Age	2019.11	53.26	<0.01
	Latitude	2,198.89	233.04	<0.01
	Latitude + Longitude + Year + Temperature + Age	2,206.79	240.94	<0.01
	Null	2,227.5	261.65	<0.01
Historic egg width	Latitude × Year	531.35	0.00	0.61
	Latitude	532.54	1.19	0.33
	Latitude + Year	537.69	6.34	0.03
	Latitude + Longitude	539.61	8.26	0.01
	Latitude × Year + Latitude	549.08	17.73	<0.01
	Latitude × Year + Latitude + Longitude	554.82	23.47	<0.01
	Null	555.28	23.93	<0.01
Historic egg length	Latitude × Year	573.43	0.00	0.9
	Latitude + Year	578.92	5.49	0.06
	Latitude	579.76	6.33	0.04
	Latitude × Year + Latitude + Longitude	584.65	11.22	<0.01
	Latitude × Year + Latitude	589.83	16.4	<0.01
	Null	612.22	38.79	<0.01
Contemporary egg width	Latitude + Longitude	1,192.26	0.00	0.43
	Latitude + Longitude + Temperature	1,193.12	0.86	0.28
	Longitude + Temperature	1,194.42	2.16	0.15
	Latitude + Longitude + Year	1,195.52	3.26	0.08
	Longitude + Year + Temperature	1,197.55	5.29	0.03
	Temperature	1,199.06	6.8	0.01
	Longitude	1,200.1	7.84	0.01
	Null	1,201.14	8.88	0.01
	Year	1,202.94	10.68	<0.01
	Latitude + Longitude + Year + Temperature + Latitude × Temperature	1,203.07	10.81	<0.01
	Latitude	1,203.36	11.1	<0.01
	Latitude + Temperature	1,204.77	12.51	<0.01
	Latitude + Year	1,205.84	13.58	<0.01
	Latitude + Year + Temperature	1,206.49	14.23	<0.01
Contemporary egg length	Latitude + Longitude	1731.57	0.00	0.6
	Latitude + Longitude + Year	1734.58	3.01	0.13
	Null	1735.36	3.79	0.09
	Year	1736.32	4.75	0.06
	Longitude	1736.87	5.3	0.04
	Latitude	1737.46	5.89	0.03
	Latitude + Longitude + Temperature	1738.45	6.88	0.02
	Latitude + Year	1739.63	8.06	0.01
	Temperature	1,740.85	9.28	0.01
	Longitude + Temperature	1741.02	9.45	0.01
	Latitude + Longitude + Year + Temperature	1741.48	9.91	<0.01
	Longitude + Year + Temperature	1743.64	12.07	<0.01
	Latitude + Temperature	1744.44	12.87	<0.01
	Latitude + Year + Temperature	1746.5	14.93	<0.01
